# Differential Contribution of Hydrogen Metabolism to *Proteus mirabilis* Fitness during Single-Species and Polymicrobial Catheterized Urinary Tract Infection

**DOI:** 10.3390/pathogens12121377

**Published:** 2023-11-22

**Authors:** Aimee L. Brauer, Brian S. Learman, Chelsie E. Armbruster

**Affiliations:** Department of Microbiology and Immunology, Jacobs School of Medicine and Biomedical Sciences, State University of New York, Buffalo, NY 14203, USA; albrauer@buffalo.edu (A.L.B.); bslearma@buffalo.edu (B.S.L.)

**Keywords:** *Proteus mirabilis*, hydrogenase, nickel, urine, urinary tract infection, *Providencia stuartii*, *Enterococcus faecalis*, polymicrobial

## Abstract

*Proteus mirabilis* is a common uropathogen and a leading cause of catheter-associated urinary tract infections (CAUTIs), which are often polymicrobial. Through a genome-wide screen, we previously identified two [NiFe] hydrogenases as candidate fitness factors for *P. mirabilis* CAUTI: a Hyb-type Group 1c H_2_-uptake hydrogenase and a Hyf-type Group 4a H_2_-producing hydrogenase. In this study, we disrupted one gene of each system (*hyfE* and *hybC*) and also generated a double mutant to examine the contribution of flexible H_2_ metabolism to *P. mirabilis* growth and fitness in vitro and during experimental CAUTI. Since *P. mirabilis* is typically present as part of a polymicrobial community in the urinary tract, we also examined the impact of two common co-colonization partners, *Providencia stuartii* and *Enterococcus faecalis*, on the expression and contribution of each hydrogenase to fitness. Our data demonstrate that neither system alone is critical for *P. mirabilis* growth in vitro or fitness during experimental CAUTI. However, perturbation of flexible H_2_ metabolism in the ∆*hybC*∆*hyfE* double mutant decreased *P. mirabilis* fitness in vitro and during infection. The Hyf system alone contributed to the generation of proton motive force and swarming motility, but only during anaerobic conditions. Unexpectedly, both systems contributed to benzyl viologen reduction in TYET medium, and disruption of either system increased expression of the other. We further demonstrate that polymicrobial interactions with *P. stuartii* and *E. faecalis* alter the expression of Hyb and Hyf in vitro as well as the contribution of each system to *P. mirabilis* fitness during CAUTI.

## 1. Introduction

Urinary tract infections (UTIs) are among the most common infections worldwide, with an estimated annual incidence of approximately 400 million cases [1]. UTIs are also the most common hospital-acquired infections, up to 80% of which are catheter-associated (CAUTI) [2]. While the majority of uncomplicated UTIs are attributed to *Escherichia coli*, the etiology of CAUTI is more diverse and includes additional and understudied uropathogens such as *Proteus mirabilis*, *Enterococcus faecalis*, *Providencia stuartii*, *Pseudomonas aeruginosa*, and *Staphylococcus* spp., especially during long-term catheterization [3]. Urine colonization and CAUTI are also frequently polymicrobial in patients catheterized for over thirty days, and we recently identified *P. mirabilis*, *P. stuartii*, and *E. faecalis* as three of the most common and persistent co-colonizing species [4].

*Proteus mirabilis* is a Gram-negative bacterium and a common cause of UTI in the elderly as well as individuals with long-term indwelling urinary catheters [5]. This organism is known for its robust swimming and swarming motility and urease activity, which contribute to persistent colonization of the urinary tract and dissemination from the bladder to the kidneys and bloodstream. *P. mirabilis* is also the most common cause of infection-induced urinary stones and a leading cause of bacteremia and mortality in catheterized individuals [6,7,8,9,10]. In addition, *P. mirabilis* isolates are intrinsically resistant to polymyxin and tetracycline-class antibiotics, often exhibit resistance to nitrofurans, fluoroquinolones, and aminoglycosides, and can acquire extended-spectrum β-lactamases and carbapenemases [5].

Several genome-wide screens have been conducted to identify genes and pathways that contribute to *P. mirabilis* colonization and persistence with the hope of revealing new potential targets for therapeutic intervention [11,12,13,14,15]. Considering that *P. mirabilis* is typically present as part of a polymicrobial community in the catheterized urinary tract [4,16], we have also begun to specifically examine the impact of polymicrobial interactions on *P. mirabilis* fitness requirements [15]. Together, these studies have revealed an important role for basic bacterial physiology and metabolism in *P. mirabilis* pathogenesis as well as infection-specific fitness requirements for survival within the urinary tract. One intriguing subset of genes that were identified through these screens pertains to the synthesis and activity of [NiFe] hydrogenases. 

[NiFe] hydrogenases catalyze the interconversion of molecular hydrogen and protons (H_2_ ↔ [H^+^ + H^−^] ↔ 2H^+^ + 2e^−^), resulting in either the production of H_2_ or the oxidization of H_2_ into protons that contribute to the proton motive force (PMF) and electrons that can enter the respiratory chain [17]. The [NiFe] hydrogenases in pathogenic bacteria have been classified into at least 13 subgroups based on properties such as their genetic organization, localization, redox partners, oxygen tolerance, and activity [17,18,19]. *P. mirabilis* HI4320 encodes two putative [NiFe] hydrogenases: a Hyb-type Group 1c hydrogenase predicted to oxidize H_2_ and generate PMF through respiration of fumarate or other high-potential oxidants [20], and a Hyf-type Group 4a hydrogenase that was recently confirmed to contribute to *P. mirabilis* acid tolerance through formate-dependent production of H_2_ [21]. 

Studies in *Salmonella* Typhimurium and *E. coli* have revealed differential regulation of multiple [NiFe] hydrogenases to provide flexibility in H_2_ metabolism, in which different enzymes contribute to aerobic respiration, anaerobic respiration, and survival during mixed acid fermentation under different redox conditions [17,22,23,24]. There is also an extensive body of work regarding the contribution of different [NiFe] hydrogenases to *Salmonella* pathogenesis (summarized in [17]). We therefore hypothesized that H_2_ production and oxidation may differentially contribute to *P. mirabilis* fitness and pathogenesis. In support of this hypothesis, two genes in the Hyf-type Group 4a hydrogenase operon (*hycI* and *hyfH*) were significantly upregulated in a mouse model of ascending UTI [14], another gene within the operon (*hyfE*) was identified as a fitness factor for bladder colonization via transposon insertion-site sequencing [15], and a third gene (*hyfG*) was shown to contribute to *P. mirabilis* colonization in a mouse model of uncomplicated UTI [21]. Furthermore, six genes in the operon were identified as fitness factors during polymicrobial CAUTI with *P. stuartii* [15]. In contrast, no genes within the Hyb-type Group 1c hydrogenase operon were upregulated during ascending UTI or identified as fitness factors for single-species CAUTI [14,15]. However, seven genes in the operon were identified as candidate fitness factors for polymicrobial CAUTI involving *P. stuartii* [15], underscoring the potential impact of the coinfection partner in the contribution to fitness. 

The goal of this study was to examine the relative contributions of the Hyb and Hyf systems to *P. mirabilis* growth under different conditions and fitness during CAUTI, as well as the impact of two common co-colonization partners (*P. stuartii* and *E. faecalis*) on the expression and contribution of both hydrogenases. Our results demonstrate that flexible H_2_ metabolism contributes to *P. mirabilis* fitness in vitro and during experimental CAUTI, and polymicrobial interactions with *P. stuartii* and *E. faecalis* alter the contribution of Hyf and Hyb to *P. mirabilis* fitness, which has implications for pursuing [NiFe] hydrogenases as therapeutic targets against *P. mirabilis*.

## 2. Materials and Methods

### 2.1. Bacterial Strains, Genome Sequencing, and Culture Conditions

*Proteus mirabilis* strain HI4320, *Providencia stuartii* strain BE2467, and *Enterococcus faecalis* strain 3143 were all previously collected from the urine of catheterized nursing home residents [25,26,27]. The complete genome sequences of *P. mirabilis* HI4320 and *P. stuartii* BE2467 are publicly available [27,28]. The genome of *E. faecalis* 3143 was sequenced as part of this study. Briefly, genomic DNA was extracted using a Qiagen DNEasy Blood and Tissue kit per the manufacturer’s instructions for Gram-positive organisms. Genomic DNA was then sent to DNA Link for library preparation via SMRTbell Express Template Prep Kit 2.0 and sequencing via PacBio Sequel II 8M SMRT cell (CLR run mode, 15 h). Sequences were submitted to the NCBI Prokaryotic Genome Annotation Pipeline (PGAP) and are available under accession number CP119528 (BioProject PRJNA941924, BioSample SAMN33617003). 

All *P. mirabilis* mutants were generated via insertion of a kanamycin resistance cassette via the Sigma TargeTron group II intron protocol [29] and verified with antibiotic selection and PCR. The hydrogenase double mutant was generated via excising the kanamycin cassette from the *hybC* mutant and disrupting *hyfE* using the TargeTron protocol with a *loxP* vector. Complemented strains were generated by cloning either *hybC* or *hyfE* along with ~500 bp flanking regions of each gene into pGen-Amp and verified with PCR as well as restoration of activity in the hydrogenase assay. 

*P. mirabilis* and *P. stuartii* were routinely cultured at 37 °C at 225 RPM in 5 mL of low-salt LB (10 g/L of tryptone, 5 g/L of yeast extract, and 0.5 g/L of NaCl). *E. faecalis* was cultured under the same conditions but in brain heart infusion (BHI) broth (Research Products International, Mount Prospect, IL, USA). To prevent *P. mirabilis* swarming, low-salt LB agar plates were generated by further decreasing the salt content to 0.1 g/L of NaCl and solidifying with 15 g/L of agar. A modified TYET medium was used in several experiments to promote hydrogenase activity (10 g/L of tryptone, 5 g/L of yeast, 50 mM of Tris, 0.4% wt/vol glucose, 30 mM of sodium formate, 1 µM of sodium selenite, and 1 µM of sodium molybdate) [30,31]. Urease activity, hydrogenase activity, and select growth curves were performed using pooled filter-sterilized human urine from at least 20 female donors (Cone Bioproducts, Seguin, TX, USA). Urine pooled from at least 5 female CBA/J mice was also used for hydrogenase activity. *Proteus mirabilis* minimal salts medium (PMSM) was used for studies requiring a defined medium (10.5 g/L of K_2_HPO_4_, 4.5 g/L of KH_2_PO_4_, 0.47 g/L of sodium citrate, and 1 g/L of (NH_4_)_2_SO_4_, supplemented with 0.001% of nicotinic acid, 1 mM of MgSO_4_, and 0.2% glycerol). Media were supplemented with kanamycin (25 µg/mL), ampicillin (25 µg/mL), and chloramphenicol (20 µg/mL) as needed for the generation and selection of *P. mirabilis* mutants. Streptomycin (150 µg/mL) was added to BHI agar plates for selection of *E. faecalis*, and chloramphenicol (20 µg/mL) was added to low-salt LB agar for selection of *P. stuartii*. For anaerobic growth experiments, cultures were incubated in anaerobic chambers with an indicator tab (BD GasPak EZ Anaerobe). 

### 2.2. Whole-Cell Hydrogenase Assay

Hydrogenase activity was assayed as previously published [30,31]. Briefly, overnight cultures of bacteria were diluted 1:100 into either fresh LB, filter-sterilized urine, or TYET. A total of 200 µL of each bacterial suspension was dispensed into the wells of a 96-well plate and incubated for 6 h at 37 °C without aeration. Then, 20 µL of a developing solution (20 mM of Tris pH 7.5, 10 mg/mL of benzyl viologen, and 250 mM of sodium formate) was added to each well, and absorbance (OD_630_) was measured every 60 s for up to 60 min using a BioTek Synergy HI. 

### 2.3. Growth Curves 

For an assessment of growth via optical density, overnight cultures of bacterial strains of interest were diluted 1:100 in a growth medium (LB, TYET, or PMSM) and distributed into replicate wells each on a clear 96-well plate. Plates were incubated at 37 °C with continuous double-orbital shaking in a BioTek Synergy H1 96-well plate reader with a 1 °C temperature differential between the top and bottom of the plate to prevent condensation. Growth was assessed via absorbance (OD_600_) at 15 min intervals for a duration of 18 h. 

For the assessment of aerobic growth via CFU quantification, overnight cultures of bacteria were diluted 1:100 in the desired growth medium (TYET or human urine) and sampled hourly for the determination of bacterial CFUs by plating onto low-salt LB agar. For anaerobic growth, cultures were incubated in anaerobic chambers with an indicator tab (BD GasPak EZ Anaerobe); a separate chamber was set up per time point, and samples were plated for the determination of CFUs at 0, 3, 6, and 24 h. The relative fitness of the hydrogenase mutants was also assessed by direct competition with wild-type *P. mirabilis* HI4320. TYET or human urine were inoculated with a 1:1 mixture of a mutant of interest and the wild-type strain, and cultures were sampled hourly for determination of bacterial CFUs by plating onto LB agar (total CFUs) and LB with kanamycin (CFUs of the mutant strain). A competitive index (CI) was calculated as follows:

$$\mathrm{CI}=\frac{\mathrm{mutant}\;\mathrm{strain}\;\mathrm{output}/\mathrm{wild}-\mathrm{type}\;P.\;mirabilisout\;\mathrm{put}}{\mathrm{mutant}\;\mathrm{strain}\;\mathrm{input}/\mathrm{wild}-\mathrm{type}\;P.\;mirabilis\;\mathrm{input}}$$

Log_10_CI = 0 indicates that the ratio of the strains in the output is similar to the input, and neither strain had an advantage; Log_10_CI > 0 indicates that the mutant has a competitive advantage over the wild type; and Log_10_CI < 0 indicates that the mutant is outcompeted by the wild type.

For polymicrobial experiments, TYET or human urine were inoculated with 1 part wild-type *P. mirabilis*, 1 part *P. mirabilis* mutant of interest, and 2 parts either *P. stuartii* or *E. faecalis*. *P. stuartii* coinfection samples were plated on plain low-salt LB (total CFUs), LB with kanamycin (*P. stuartii* + *P. mirabilis* mutant CFUs), and LB with chloramphenicol (*P. stuartii* CFUs). *E. faecalis* coinfection samples were plated on plain low-salt LB (total CFUs), LB with kanamycin (*P. mirabilis* mutant + *E. faecalis* CFUs), and BHI with streptomycin (*E. faecalis* CFUs). 

### 2.4. Acid Tolerance Assay

Overnight cultures of strains of interest were diluted 1:50 in either LB or TYET and incubated at 37 °C with aeration for 2–3 h until log phase. Cultures were then centrifuged to pellet, resuspended in 10 mM of 2-(*N*-morpholino)-ethanesulfonic acid (MES; Sigma) buffer to an OD_600_ of ~1.0, and diluted 1:10 in 1 mL of 10 mM MES adjusted to pH 3, 5, or 7 with HCl. Samples were incubated at 37 °C with aeration for 1 h and plated using an EddyJet 2 spiral plater (Neutec Group, Farmingdale, NY, USA) for the determination of CFUs using a ProtoCOL 3 automated colony counter (Synbiosis, Frederic, MD, USA).

### 2.5. Motility Assays

Swarming motility was assessed by inoculating 5 µL of an overnight culture of *P. mirabilis* HI4320 or isogenic mutant onto the surface of a swarm agar plate (LB agar made with 5 g/L of NaCl), allowing the inoculum to soak in for ~10 min, and incubating at 37 °C for 18 h prior to measurement of the diameter of each swarm ring. Swarm plates were either incubated under aerobic conditions or incubated in anaerobic chambers with an indicator tab (BD GasPak EZ Anaerobe).

### 2.6. Urease Assay

Urease activity was measured as described previously [27]. Overnight cultures of strains of interest were diluted 1:50 in either LB or TYET and incubated at 37 °C with aeration for 2–3 h until log phase. Cultures were then centrifuged to pellet, adjusted to 10^9^ CFU/mL, and diluted 1:10 in filter-sterilized human urine containing 0.001% wt/vol phenol red and 500 mM of urea and dispensed into a clear-bottom 96-well plate. Absorbance (OD_562_) was measured every 30 s for a total of 90 min using a BioTek Synergy HI. 

### 2.7. RNA Extraction and qRT-PCR

Samples for RNA extraction were prepared by inoculating 6 mL of TYET media with bacteria of interest and collecting at 0, 2, and 6 h time points. At each time point, the culture was centrifuged for 20 min at 8000 rcf, the supernatant was removed, and the pellet was resuspended in 50 µL of RLT Buffer with BME (Qiagen RNeasy Mini Kit, Hilden, Germany). Then, 1.5 mL safe-lock microfuge tubes (Eppendorf, Hamburg, Germany) were filled halfway with 0.5 mm of glass disruption beads (RPI Research Products International), the suspended pellet was added to the bead tube, and the samples were homogenized using a Bullet Blender Gold (Next Advance, Troy, NY, USA) at max speed for 5 min. After bead beating, 250 µL of RLT Buffer with BME was added, and samples were vortexed to mix. A small hole was then punched in the bottom of the tube with an 18 G needle (BD Precision Glide), and the tube was immediately placed inside a 1.5 mL microfuge tube to collect the homogenate while leaving the beads behind. The tube containing the beads was discarded, and the homogenate was centrifuged at 21,000 rcf for 10 min at 4 °C. The supernatant was removed, and 1 volume of 70% ethanol was added and vortexed to mix. The sample was then transferred to an RNeasy Mini spin column, placed in a 2 mL collection tube, and centrifuged for 15 s at 8000 rcf. The flow through was transferred back to the column and centrifuged again for maximum binding. Then, 350 µL of RWI buffer was added to the column and centrifuged for 15 s at 8000 rcf. DNA was then digested using an on-column DNase digestion kit (Qiagen), in which 80 µL of DNase I was applied directly to the column and incubated at room temperature for 25 min. After, 350 µL of RWI buffer was added to the column and centrifuged for 15 s at 8000 rcf. The column was washed twice with 500 µL of Buffer RPE. The column was then placed in a new 1.5 mL tube, and 40 µL of RNase-free water was added directly to the column, then centrifuged for 1 min at 8000 rcf. A second DNase digestion was then performed off-column by adding 0.1 volume of 10X DNase and 1 µL of DNase (Invitrogen, Carlsbad, CA, USA) and incubating at 37 °C for 30 min. The digestion was terminated by adding 0.1 volumes of DNase inactivation reagent, incubating at room temperature for 2 min, and centrifuging for 90 s at 8000 rcf. cDNA was synthesized using the iScript cDNA Synthesis Kit (BioRad, Hercules, CA, USA), and qRT-PCR was performed as previously described [31] using qPCRBio SyGreen Blue Mix Lo-Rox (PCR Biosystems, London, UK) and a BioRad CFX-Connect Real Time system. Data were normalized to *rpoA* as the reference gene as it exhibited low variation between strains. Data were analyzed according to the Relative Quantification (RQ) method, in which the time-0 sample for each strain/condition was considered the “Control” and the 4 and 6 h samples were considered the “Sample” in the following equation:

$$\mathrm{Fold}\;\mathrm{Change}=\frac{{\left(E_{\mathrm{target}}\right)}^{\Delta \mathrm{CPtarget}\;(\mathrm{control}-\mathrm{sample})}}{{\left(E_{\mathrm{reference}}\right)}^{\Delta \mathrm{CPrefernce}\;(\mathrm{control}-\mathrm{sample})}}$$

In this equation, *E* refers to the primer efficiency of the target gene (*E*_target_) or the reference gene (*E*_reference_), and CP refers to the Cycle Point, or the cycle number at which the signal exceeds the threshold.

### 2.8. Mouse Model of CAUTI

CAUTI co-challenge studies were carried out as previously described using female CBA/J mice aged 6–8 weeks (Jackson Laboratory) [15]. Briefly, the bacterial inoculum was prepared by washing overnight cultures of *P. mirabilis* and mutants in phosphate-buffered saline (PBS: 0.128 M of NaCl, 0.0027 M of KCl, and pH 7.4), adjusting to OD_600_ 0.2 (~2 × 10^8^ CFU/mL), mixing the desired strains 1:1, and diluting 1:100 to achieve an inoculum of 2 × 10^6^ CFU/mL. Mice were anesthetized with a weight-appropriate dose (0.1 mL for a mouse weighing 20 gm) of ketamine/xylazine (80–120 mg/kg ketamine and 5–10 mg/kg xylazine) via IP injection and inoculated transurethrally with 50 µL of the diluted suspensions as designated in the text (1 × 10^5^ CFU/mouse), and a 4 mm segment of sterile silicone tubing (0.64 mm O.D., 0.30 mm I.D., Braintree Scientific, Inc., Braintree, MA, USA) was placed in the bladder during inoculation as previously described [27,32]. At 96 h post-inoculation (hpi), urine was collected, mice were euthanized, and bladders, kidneys, and spleens were harvested into 5 mL Eppendorf tubes containing 1 mL of PBS. Tissues were homogenized using a Bullet Blender 5 Gold (Next Advance) and plated using an EddyJet 2 spiral plater (Neutec Group, Farmingdale, NY, USA) onto plain low-salt LB agar (total CFUs) and low-salt LB with kanamycin (mutant CFUs) for quantification using a ProtoCOL 3 automated colony counter (Synbiosis). A competitive index was calculated for each co-challenge as described above for urine growth curves.

For polymicrobial cochallenge experiments, mice were inoculated with a mixture containing 2.5 × 10^4^ CFUs of the indicated mutant, 2.5 × 10^4^ CFUs of wild-type *P. mirabilis*, and 5 × 10^4^ CFUs of the other species. *P. stuartii* coinfection samples were plated on plain low-salt LB (total CFUs), LB with kanamycin (*P. stuartii* + *P. mirabilis* mutant CFUs), and LB with chloramphenicol (*P. stuartii* CFUs). *E. faecalis* coinfection samples were plated on plain low-salt LB (total CFUs), LB with kanamycin (*P. mirabilis* mutant + *E. faecalis* CFUs), and BHI with streptomycin (*E. faecalis* CFUs). 

### 2.9. Statistical Analysis

Significance was assessed using two-way analysis of variance (ANOVA) corrected for multiple comparisons, the Student’s *t* test, the Wilcoxon signed-rank test, and the one-sample *t* test, as indicated in the figure legends. All analyses were performed using GraphPad Prism, version 7.03 (GraphPad Software). All *p* values are two-tailed at a 95% confidence interval. 

## 3. Results

### 3.1. Genomic Context of the Two [NiFe] Hydrogenases in P. mirabilis HI4320

**Figure 1:**
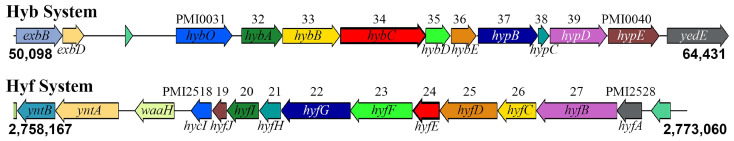
**Genomic context of the Hyb-type and Hyf-type [NiFe] hydrogenases of *P. mirabilis*.** *Proteus mirabilis* strain HI4320 encodes two putative [NiFe] hydrogenases: a Hyb-type Group 1c hydrogenase (*hybOABCDE* and four associated maturation factors, *hypBCDE*, PMI0031-0040) and a Hyf-type Group 4a formate hydrogenlyase (*hyfABCDEFGHIJ* and the maturation factor *hycI*, PMI2518-2528). Each operon display comes from the Compare Region Viewer available through the Bacterial and Viral Bioinformatics Resource Center (BV-BRC) database and includes the genes flanking each operon. Numbers indicate nucleotide positions within the HI4320 genome.

The Hyb-type Group 1c hydrogenase (*hybOABCDE* with four associated maturation factors, *hypBCDE*, PMI0031-0040) and the Hyf-type Group 4a formate hydrogenlyase (*hyfABCDEFGHIJ* with the maturation factor *hycI*, PMI2518-2528) are displayed in Figure 1. The *hyb* operon is flanked by genes encoding the putative TonB partners *exbBD* (PMI0029-30) and two putative factors involved in selenium metabolism (*yedEF* PMI0041-42), while the *hyf* operon is adjacent to the operon encoding the Ynt nickel ABC transport system that we previously identified as the primary nickel importer for both urease activity and hydrogenase activity in this strain [31]. None of the genes in either hydrogenase operon were estimated to be essential for the growth of *P. mirabilis* in LB broth, as transposon mutants in all genes were well represented in our *P. mirabilis* transposon mutant libraries [15].

In order to examine the contribution of each system to *P. mirabilis* fitness, we selected one gene from each operon that exhibited 100% amino acid identity across all 97 *P. mirabilis* isolates with complete genome sequences from the Bacterial and Viral Bioinformatics Resource Center (BV-BRC) database and was representative of our prior transposon insertion-site sequencing screens. We therefore chose the gene encoding the large subunit of the Hyb system (*hybC*) and one of the membrane components of the Hyf system (*hyfE*) for disruption to examine their contribution to *P. mirabilis* growth, fitness, and polymicrobial interactions. 

### 3.2. HybC and HyfE Both Contribute to Benzyl Viologen Reduction by P. mirabilis HI4320

**Figure 2:**
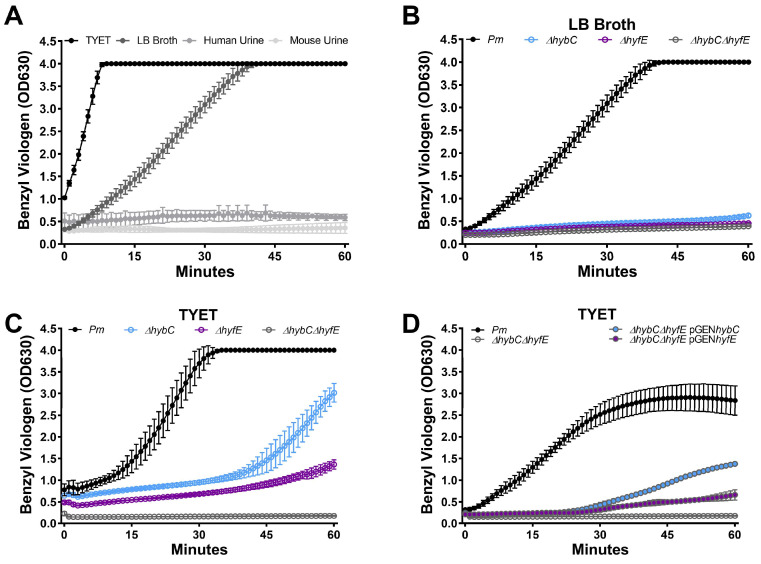
**HybC and HyfE are both important for benzyl viologen reduction via *P. mirabilis*.** (**A**) *P. mirabilis* was cultured in LB broth overnight, washed once in PBS, and diluted 1:100 into either TYET medium, LB broth, pooled human urine, or pooled mouse urine. Each bacterial suspension was incubated for 6 h at 37 °C without aeration, followed by colorimetric quantification of hydrogenase activity via the benzyl viologen reduction (read at OD_630_). (**B**,**C**) Benzyl viologen reduction via wild-type *P. mirabilis* and hydrogenase mutants after incubation in LB broth (**B**) or TYET (**C**). (**D**) Benzyl viologen reduction via wild-type *P. mirabilis* and the ∆*hybC*∆*hyfE* double mutant complemented with an empty vector (pGEN), *hybC* (pGEN*hybC*), or *hyfE* (pGEN*hyfE*) after incubation in TYET. Graphs are representative of four independent experiments; error bars indicate mean and standard deviation (SD) for three technical replicates.

Hydrogenase activity of strain HI4320 was first assessed using a whole-cell assay that measures activity via benzyl viologen reduction [30] that we previously utilized to examine the contribution of two nickel import systems to hydrogenase activity in *P. mirabilis* [31]. Wild-type *P. mirabilis* was incubated for 6 h in either TYET (a medium with tryptone, yeast extract, glucose, formate, sodium molybdate, and sodium selenite that was previously shown to support Group 1c and Group 4a [NiFe] hydrogenase activity [30]), LB broth, filter-sterilized human urine, or filter-sterilized mouse urine. A robust and rapid color change from clear to purple (indicating benzyl viologen reduction) was observed in both TYET and LB broth, but no color change was detected in either human or mouse urine (Figure 2A). We therefore chose to utilize TYET and LB to examine the contribution of *hybC* and *hyfE*. Disruption of either *hybC* or *hyfE* completely abrogated the color change in LB broth (Figure 2B) and substantially delayed the color change in TYET (Figure 2C), indicating that both [NiFe] hydrogenases can contribute to benzyl viologen reduction under these experimental conditions. Hyb and Hyf also appear to be the only systems capable of mediating the color change under these experimental conditions, as no color change was detected for a Δ*hybC*Δ*hyfE* double mutant in either LB or TYET (Figure 2B,C). Importantly, complementation of the Δ*hybC*Δ*hyfE* double mutant with either *hybC* or *hyfE* on a plasmid restored a similar amount of benzyl viologen reduction as observed for the single mutants (Figure 2D), confirming that loss of activity is specifically due to disruption of *hybC* and *hyfE*. 

### 3.3. H_2_ Metabolism Is Not Required for P. mirabilis Growth under Standard In Vitro Conditions

**Figure 3:**
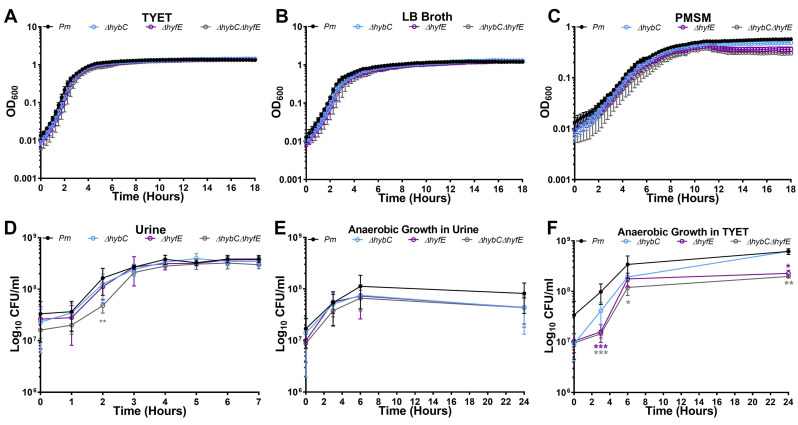
**HybC and HyfE are not required for optimal growth of *P. mirabilis*.** (**A**–**C**) Aerobic growth was assessed with automated measurement of OD_600_ at 15-minute intervals in a plate reader for bacteria suspended in TYET (**A**), LB broth (**B**), or *Proteus* minimal salts medium (PMSM) (**C**). Error bars represent mean ± SD of at least 3 replicates, and graphs are representative of 3 independent experiments. No significant differences were detected using a two-way ANOVA. (**D**–**F**) Growth was assessed by plating for viable CFUs at hourly intervals for bacteria incubated aerobically in pooled human urine (**C**), anaerobically in pooled human urine (**D**), or anaerobically in TYET (**E**). Anaerobic conditions were established in GasPak chambers with an indicator tab. Error bars represent mean ± SD of 3 independent experiments with 3 replicates each. * *p* < 0.05, ** *p* < 0.01, and *** *p* < 0.001 using two-way ANOVA.

To examine the contribution of flexible H_2_ metabolism to *P. mirabilis* growth in vitro, wild-type *P. mirabilis* and mutants were cultured aerobically in TYET, LB broth, and *Proteus* minimal salts medium (PMSM) for 18 h (Figure 3A–C). No differences in growth rate were observed for any of the mutants compared to wild-type *P. mirabilis*, indicating that *hybC* and *hyfE* are not required for optimal aerobic growth in vitro under these standard conditions. Growth was also assessed in filter-sterilized human urine over 7 h (Figure 3D). The Δ*hybC*Δ*hyfE* double mutant displayed a slight decrease in CFUs at the 2 h timepoint compared to the wild type, but all strains reached equivalent density by 3 h and maintained similar viability throughout the rest of the time course, indicating that *hybC* and *hyfE* are not critical for aerobic growth in urine in vitro. Considering that loss of [NiFe]-hydrogenase activity in *E. coli* only results in a growth defect under anaerobic conditions [33,34,35], we also assessed viability during anaerobic growth in human urine (Figure 3E) and TYET (Figure 3F). No significant differences were detected in human urine, but *hyfE* was required for optimal anaerobic growth in TYET, as both the Δ*hyfE* and Δ*hybC*Δ*hyfE* double mutants exhibited reduced CFUs compared to the wild type. Notably, the Δ*hyfE* and Δ*hybC*Δ*hyfE* double mutants grew similarly under this condition, indicating that the Hyb Group 1c [NiFe] likely does not contribute to *P. mirabilis* growth in TYET under these conditions.

### 3.4. The Hyf-Type Group 4a [NiFe] Hydrogenase Contributes to PMF under Anaerobic Conditions

Group 1c and Group 4a [NiFe] hydrogenases from other species such as *E. coli* and *Salmonella* Typhimurium are thought to contribute to proton motive force (PMF) under certain conditions [17,18,19]. In *P. mirabilis*, Lin et al. reported that disruption of *hyfG* in strain N2 decreased acid tolerance under aerobic conditions [21], which we have found to be a reliable surrogate for assessing alterations in membrane potential and PMF [36,37]. However, the decrease was minor (~1% survival compared to ~6% survival for wild-type *P. mirabilis*), and loss of *hyfG* did not alter swarming motility under aerobic conditions [21], which represents another phenotype linked to membrane potential as flagellar rotation requires PMF [38]. Thus, the contribution of the Group 4a Hyf system to PMF generation and motility requires further study in *P. mirabilis.* The Group 1c Hyb system was also previously reported to contribute to *P. mirabilis* HI4320 swarming motility under anaerobic conditions [17,39]. However, the *hybB* transposon mutant that was screened in that study (25H1) was actually demonstrated to achieve the same total swarm diameter as wild-type *P. mirabilis* HI4320 under both aerobic and anaerobic conditions [39], indicating that the contribution of the Group 1c Hyb system to PMF similarly requires further study. We therefore sought to determine the contribution of each hydrogenase to both acid tolerance and swarming motility. 

**Figure 4:**
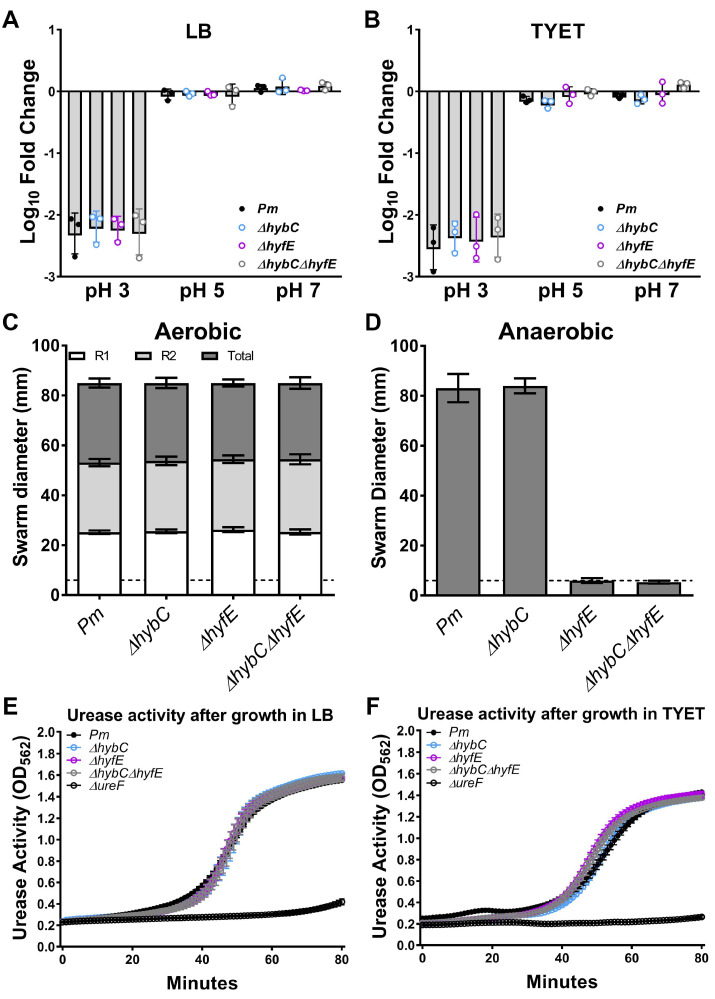
**HybC and HyfE do not contribute to acid tolerance, motility, or urease activity of *P. mirabilis* HI4320, but HyfE contributes to PMF under anaerobic conditions.** (**A**,**B**) Bacteria were cultured to mid-log in either LB (**A**) or TYET (**B**), washed, resuspended in buffer at pH 3, 5, or 7, and incubated for 60 min prior to plating for viability. The data display the log_10_ fold change in viability compared to the inoculum; error bars represent the mean ± SD of 3 independent experiments. No significant differences were detected using two-way ANOVA. (**C**,**D**) Bacteria were cultured overnight, spotted onto the surface of a swarm agar plate, and incubated overnight at 37 °C under aerobic (**C**) or anaerobic (**D**) conditions. Following incubation, the diameter of each visible swarm ring as well as total distance migrated were measured. Error bars represent the mean ± SD of 3 independent experiments with 3 replicates each. (**E**,**F**) Bacteria were cultured overnight in LB broth, sub-cultured in either LB (**A**) or TYET (**B**) to mid-log phase, washed, and resuspended in filter-sterilized human urine supplemented with 500 mM urea and phenol red. Urease activity was assessed via a colorimetric assay at 37 °C in a 96-well plate reader via measurement of absorbance (OD_562_) at 30 s intervals for 80 min. Error bars represent the mean ± SD of at least 3 replicates, and graphs are representative of 3 independent experiments. No significant differences were detected using two-way ANOVA.

For acid tolerance experiments, wild-type *P. mirabilis* and mutants were grown to mid-log phase in either LB or TYET, washed, and resuspended in buffer at pH 3, 5, or 7, and viability was assessed after 60 min. Consistent with our prior studies, incubation at pH 3 resulted in a >2 log reduction in *P. mirabilis* viability, while incubation at pH 5 or 7 had minimal impact (Figure 4A,B). However, no differences were observed for any of the hydrogenase mutants, indicating that neither [NiFe] hydrogenase contributes to acid tolerance in strain HI4320 under these conditions. We next assessed swarming motility for wild-type *P. mirabilis* and each mutant when incubated under aerobic or anaerobic conditions (Figure 4C,D). All of the mutants exhibited similar swarming patterns and total diameter as wild-type *P. mirabilis* under aerobic conditions, but the Δ*hyfE* mutant and Δ*hybC*Δ*hyfE* double mutant were completely inhibited for swarming motility under anaerobic conditions. We therefore conclude that the Hyb system does not appear to contribute to membrane potential or PMF in strain HI4320 when grown on LB or a TYET medium, but the Hyf system contributes to PMF on LB under anaerobic conditions. 

Lin et al. also previously demonstrated that the Hyf-type system of *P. mirabilis* contributes to optimal urease activity [21]. Specifically, disrupting *hyfG* in *P. mirabilis* strain N2 decreased urease activity in synthetic urine. We therefore assessed the urease activity of Δ*hybC*, Δ*hyfE*, and Δ*hybC*Δ*hyfE* in pooled human urine compared to wild-type *P. mirabilis* HI4320 and a urease-negative mutant (Δ*ureF*) using our whole-cell urease assay [40]. Wild-type *P. mirabilis* and each mutant were grown to mid-log phase in either LB or a TYET medium to induce hydrogenase activity, then washed and resuspended in human urine for measurement of urease activity. Surprisingly, all hydrogenase mutants exhibited comparable urease activity to wild-type *P. mirabilis* when incubated in pooled human urine, regardless of whether they were initially grown to mid-log phase in LB broth or a TYET medium (Figure 4C,D). Thus, neither system appears to contribute to urease activity in *P. mirabilis* strain HI4320 in human urine. This discrepancy could be due to differences in the composition of synthetic urine compared to human urine, especially since neither hydrogenase system appears to be active during aerobic growth in human urine (Figure 2A). Additional potential explanations for the discrepancy are the methods used for monitoring urease activity, strain differences, and a difference in which component of the hydrogenase system was inactivated (*hyfE* versus *hyfG*). 

### 3.5. HybC and HyfE Are Both Highly Induced during Growth in the TYET Medium

**Figure 5:**
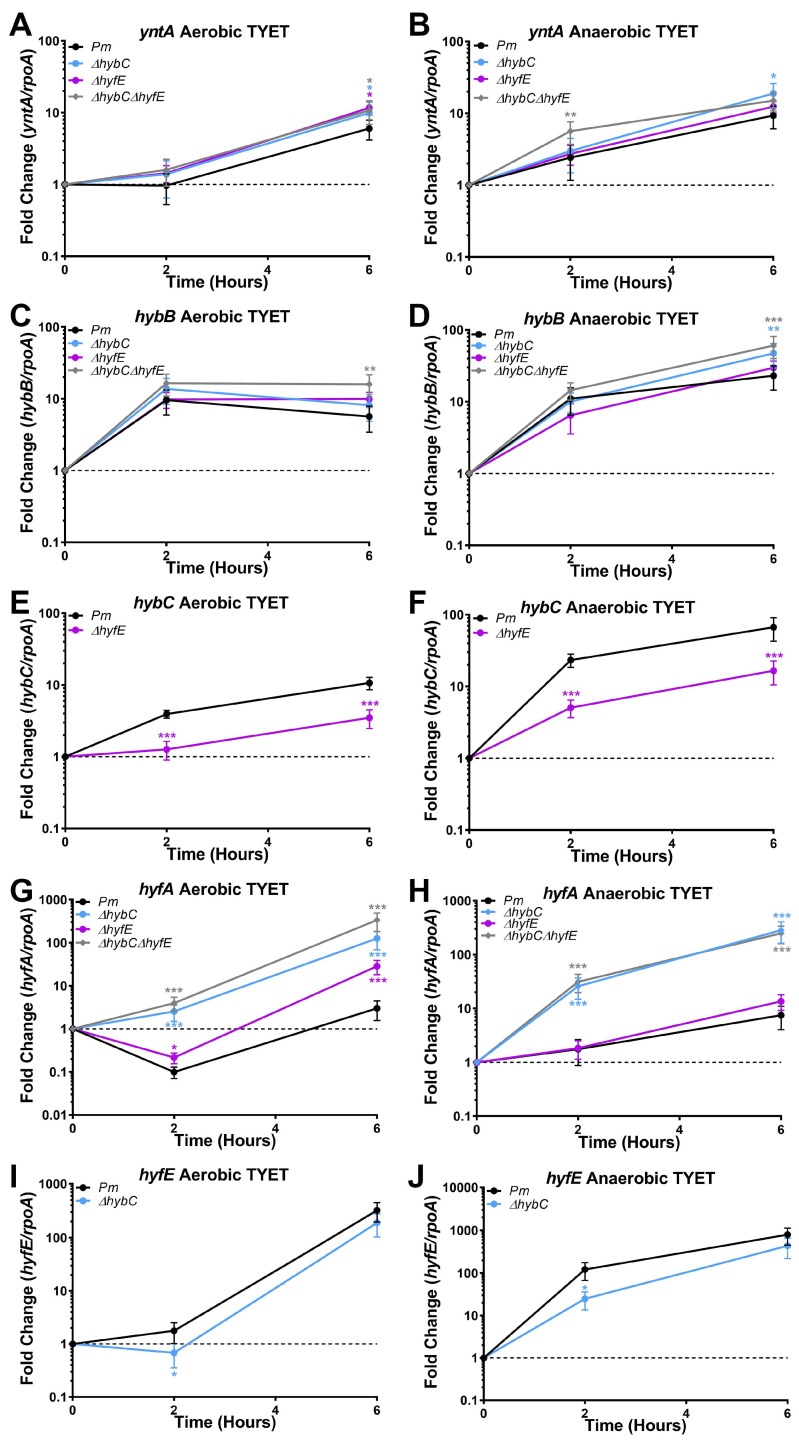
**The Hyb and Hyf systems are highly expressed with *P. mirabilis* during growth in the TYET medium.** The TYET medium was inoculated with wild-type *P. mirabilis* or indicated mutants and incubated for 6 h at 37 °C under aerobic conditions or anaerobic conditions in GasPak chambers with an indicator. RNA was isolated at 0, 2, and 6 h post-inoculation for cDNA synthesis and qRT-PCR. The Pfaffl method was utilized to determine the fold change in expression of *yntA* (**A**,**B**), *hybB* (**C**,**D**), *hybC* (**E**,**F**), *hyfA* (**G**,**H**), and *hyfE* (**I**,**J**) over time for each strain compared to expression at time 0, with normalization to the housekeeping gene *rpoA* and accounting for differences in primer efficiencies. Error bars represent the mean and standard deviation for three independent experiments with two technical replicates each. * p < 0.05, ** p < 0.01, and *** p < 0.001 using two-way ANOVA compared to wild-type *P. mirabilis*.

Our data thus far indicate that H_2_ metabolism is not critical for *P. mirabilis* growth in vitro under aerobic or anaerobic conditions, but Hyf-type Group 4a hydrogenase activity contributes to PMF under anaerobic conditions. Since both hydrogenases contributed to benzyl viologen reduction in the TYET medium and there were differences in the kinetics of reduction between the Δ*hybC* and Δ*hyfE* mutants, we further examined the expression profiles of each system during growth in the TYET medium. Wild-type *P. mirabilis* and all mutants were incubated in TYET and aerobic or anaerobic conditions, and samples were collected at 0, 2, 4, and 6 h post-inoculation for qRT-PCR (Figure 5). We were specifically interested in mRNA levels of the substrate binding protein for the primary nickel importer in *P. mirabilis* HI4320 (*yntA*), the first membrane subunit in the *hyb* operon (*hybB*), *hybC*, the first gene in the *hyf* operon (*hyfA*), and *hyfE.* Expression was normalized to the housekeeping gene *rpoA* at each time point, and fold change is reported relative to expression at time 0 for each strain. Expression of *yntA* followed a time-dependent increase in all strains regardless of culture condition (Figure 5A,B), suggesting increased nickel import to fuel hydrogenase activity. Interestingly, *yntA* expression was slightly increased in all of the hydrogenase mutants compared to the wild type under aerobic conditions (Figure 5A) and in the Δ*hybC* and Δ*hybC*Δ*hyfE* mutants under anaerobic conditions (Figure 5B), which might suggest increased demand for nickel import. 

Regarding the Hyb-type Group 1c hydrogenase, a time-dependent increase in expression of *hybB* and *hybC* was observed regardless of culture condition (Figure 5C,D). Expression of *hybB* exhibited a slight, though statistically significant, increase at 6 h in the Δ*hybC*Δ*hyfE* double mutant under aerobic conditions and both the Δ*hyfC* and Δ*hybC*Δ*hyfE* double mutant under anaerobic conditions, but no differences were observed for the Δ*hyfE* mutant. In contrast, *hybC* expression was substantially decreased in the Δ*hyfE* mutant under both conditions (Figure 5E,F), suggesting differential regulation of *hybB* and *hybC* mRNA levels and revealing a clear impact of disrupted Hyf-type Group 4a hydrogenase activity on expression of the Hyb-type Group 1c hydrogenase. 

For the Hyf-type Group 4a hydrogenase, clear differences were also observed in the expression patterns of *hyfA* and *hyfE*. Under aerobic conditions, expression of *hyfA* initially decreased in wild-type *P. mirabilis* before increasing ~3-fold above baseline (Figure 5G), while expression of *hyfE* exhibited a time-dependent increase (Figure 5I). Under anaerobic conditions, both *hyfA* and *hyfE* exhibited a time-dependent increase in wild-type *P. mirabilis* but of different magnitudes; *hyfA* was increased ~7.5-fold over baseline by 6 h, while *hyfE* was increased ~800-fold over baseline (Figure 5H,J). There were also noticeable differences in the impact of disrupted H_2_ metabolism on *hyfA* and *hyfE* expression. The Δ*hyfE* mutant largely followed the same *hyfA* expression profile as wild-type *P. mirabilis* under both aerobic and anaerobic conditions, although expression declined to a lesser degree than wild-type at 2 h and then increased ~30-fold above baseline by 6 h. However, *hyfA* expression in both the Δ*hyfC* and Δ*hybC*Δ*hyfE* mutants was dramatically higher than the level of expression achieved with either wild-type *P. mirabilis* or the Δ*hyfE* mutant (Figure 5G,H). This observation strongly suggests that disrupting Hyb-type Group 1c hydrogenase activity impacts the expression of Hyf-type Group 4a hydrogenase. In contrast, *hyfE* expression was slightly lower in the Δ*hyfC* mutant compared to wild-type *P. mirabilis*, suggesting differential regulation of *hyfA* and *hyfE* in both wild-type *P. mirabilis* and the Δ*hyfC* mutant. Taken together, these data demonstrate that genes of both the Hyb-type and Hyf-type [NiFe] hydrogenases are upregulated using *P. mirabilis* during growth in the TYET medium and that disruption of one system impacts expression of the other. 

### 3.6. [NiFe] Hydrogenase Activity Contributes to Competitive Fitness during Growth in the TYET Medium

**Figure 6:**
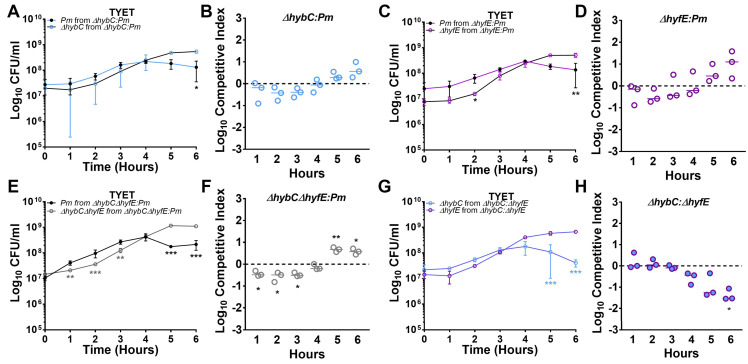
**HybC and HyfE contribute to *P. mirabilis* fitness in TYET medium.** TYET medium was inoculated with a 1:1 mixture of ∆*hybC* and wild-type *P. mirabilis* (**A**,**B**), ∆*hyfE* and wild-type *P. mirabilis* (**C**,**D**), ∆*hybC*∆*hyfE* and wild-type *P. mirabilis* (**E**,**F**), or ∆*hybC* and ∆*hyfE* (**G**,**H**) and CFUs were quantified at hourly intervals. A competitive index was calculated using the ratio of the indicated strains at each time point compared to the ratio at time = 0 (panels **B**,**D**,**F**,**H**). Data represent the mean ± SD of 3 independent experiments. Significant differences in the competitive index were determined with Wilcoxon signed rank and in growth curves using two-way ANOVA. * p < 0.05, ** *p* < 0.01, and *** *p* < 0.001.

While none of the mutants exhibited pronounced defects during growth in vitro, the possibility remains that each hydrogenase may contribute to the overall fitness of *P. mirabilis*, which can be assessed by directly competing a mutant against its parental strain. Competitive challenge experiments were therefore conducted to examine the contribution of H_2_ metabolism to *P. mirabilis* fitness during aerobic growth in TYET. The ∆*hybC* mutant and wild-type *P. mirabilis* exhibited comparable CFUs for the first 4 h of co-culture, but wild-type *P. mirabilis* CFUs plateaued at this point and then slightly declined while ∆*hybC* CFUs increased and reached stationary phase (Figure 6A). A competitive index was calculated by examining the ratio of the indicated strains at each time point compared to the ratio at time zero, which revealed a slight trend towards increased fitness of the *hybC* mutant by 5 h (*p* = 0.065, Figure 6B). A similar trend was observed for the *hyfE* mutant, which exhibited slightly decreased CFUs than wild-type *P. mirabilis* during early time points, followed by a decrease in wild-type CFUs at later time points (Figure 6C,D). This trend was further amplified in the Δ*hybC*Δ*hyfE* double mutant, which had a fitness defect at early time points but then caught up and outcompeted wild-type *P. mirabilis* by early stationary phase (Figure 6E,F). Thus, both [NiFe] hydrogenases appear to provide a slight fitness advantage to *P. mirabilis* during the lag phase and early log phase in the TYET medium, but the high level of expression and activity in the wild type appears to become detrimental as the bacteria reach the stationary phase in this growth medium. 

We next sought to determine if one hydrogenase system was dominant over the other by directly competing the *hybC* and *hyfE* mutants in the absence of wild-type *P. mirabilis.* Both mutants achieved comparable CFUs in TYET for the first 3 h, but Δ*hybC* CFUs plateaued at this point and then declined while ∆*hyfE* CFUs increased and reached stationary phase (Figure 6G). The competitive index calculation also clearly showed that Δ*hybC* was outcompeted by ∆*hyfE* during the co-challenge (Figure 6H). Thus, either the decreased expression and activation of the Hyb system in the ∆*hyfE* mutant provides an overall fitness advantage in the TYET medium or the increased expression and activation of the Hyf system in the Δ*hybC* mutant causes a fitness defect. 

### 3.7. Flexible H_2_ Metabolism Contributes to Competitive Fitness during Experimental CAUTI

**Figure 7:**
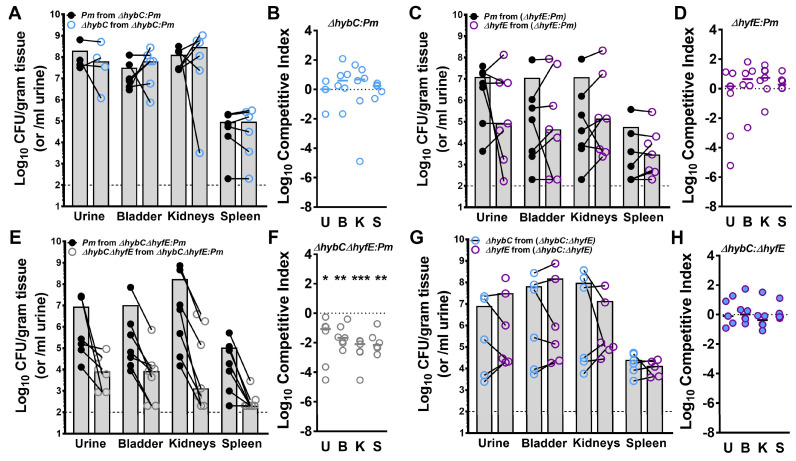
**Flexible H_2_ metabolism contributes to *P. mirabilis* fitness within the catheterized urinary tract.** Bacteria were cultured in LB overnight, washed once in PBS, and adjusted to 2 × 10^6^ CFU/mL. Female CBA/J mice were transurethrally inoculated with 50 µL of a 1:1 mix of ∆*hybC* and wild-type *P. mirabilis* (**A**,**B**), ∆*hyfE* and wild-type *P. mirabilis* (**C**,**D**), ∆*hybC*∆*hyfE* and wild-type *P. mirabilis* (**E**,**F**), or ∆*hybC* and ∆*hyfE* (**G**,**H**). During inoculation, a 4 mm segment of sterile silicone catheter tubing was also inserted into the bladder of each mouse to mimic CAUTI. (**A**,**C**,**E**,**G**) Each symbol represents the Log_10_ CFU per milliliter of urine or gram of tissue from an individual coinfected mouse, with the CFUs of each bacterial strain connected with a black line. Gray bars represent the mean, and the dashed line indicates the limit of detection. (**B**,**D**,**F**,**H**) A competitive index was calculated based on the ratio of each strain in each organ divided by the ratio of each strain in the input inoculum. Each symbol represents the Log_10_ CI for an individual mouse, error bars represent the median, and the dashed line indicates Log_10_ CI = 0 (the expected value if the ratio is 1:1). * p < 0.05, ** p < 0.01, and *** p < 0.001 with the Wilcoxon signed-rank test.

To assess the contribution of [NiFe] hydrogenase activity and flexible H_2_ metabolism to *P. mirabilis* fitness within the catheterized urinary tract, female CBA/J mice were transurethrally inoculated with 10^5^ CFUs of a 50:50 mixture of wild-type *P. mirabilis* HI4320 and either ∆*hybC*, ∆*hyfE*, or the ∆*hybC*∆*hyfE* double mutant. A 4 mm segment of sterile catheter tubing was placed in the bladder during inoculation, and mice were euthanized 96 h post-inoculation for determination of bacterial burden. The ∆*hybC* and ∆*hyfE* single mutants were outcompeted by wild-type *P. mirabilis* in a few individual mice, but no significant fitness defects were detected with the competitive index calculation (Figure 7A–D). However, disrupting control of flexible H_2_ metabolism in the ∆*hybC*∆*hyfE* double mutant resulted in a severe fitness defect in all organs of all mice (Figure 7E,F). When the ∆*hybC* and ∆*hyfE* mutants were directly competed against each other, they exhibited comparable colonization and fitness (Figure 7G,H). Thus, *P. mirabilis* fitness during experimental CAUTI appears to be maintained as long as at least one hydrogenase system remains intact, but the complete loss of H_2_ metabolism results in a severe defect. Importantly, these data demonstrate an infection-specific contribution of the hydrogenases to fitness, as no defects were observed during incubation in urine in vitro (Supplemental Figure S1). 

### 3.8. Polymicrobial Interactions Alter the Expression and Contribution of [NiFe] Hydrogenases to P. mirabilis Fitness in TYET Medium

*P. mirabilis* is frequently found as part of a polymicrobial community in the catheterized urinary tract, and our prior studies demonstrated that two common co-colonizing partners, *Providencia stuartii* and *Enterococcus faecalis*, substantially increase *P. mirabilis* pathogenic potential and CAUTI severity [27,40]. We also previously determined that coinfection with *P. stuartii* alters the genes and pathways that *P. mirabilis* requires for colonization of the catheterized urinary tract [15]. We therefore sought to determine the impact of polymicrobial interactions on the contribution of each hydrogenase system to *P. mirabilis* fitness. The genome sequence of *P. stuartii* BE2467 includes a single putative [NiFe] hydrogenase belonging to Hyb-type Group 1c (BGK56_21290-21325) for which *hybC* exhibits 72% amino acid identity to *P. mirabilis*. In contrast, the genome of *E. faecalis* 3143 lacks all known [NiFe] hydrogenase systems. 

**Figure 8:**
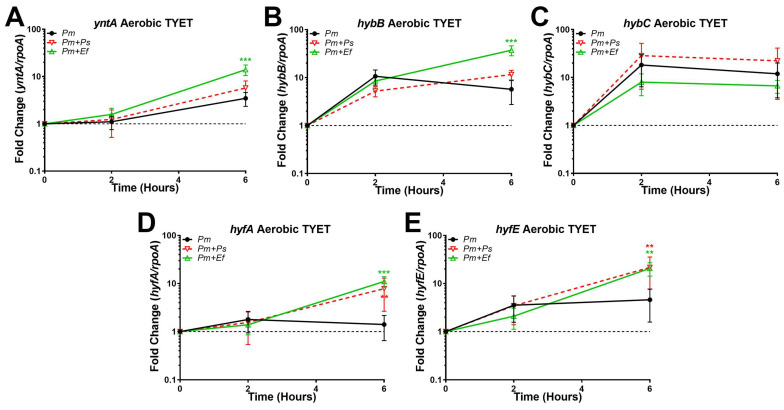
**Polymicrobial interactions alter the expression of the Hyb and Hyf systems in TYET medium.** TYET medium was inoculated with wild-type *P. mirabilis* alone or a 1:1 mixture of *P. mirabilis* with either *E. faecalis* or *P. stuartii* and incubated for 6 h at 37 °C under aerobic conditions. RNA was isolated at 0, 2, and 6 h post-inoculation for cDNA synthesis and qRT-PCR, and the fold change in gene expression over time was assessed as above for *yntA* (**A**), *hybB* (**B**), *hybC* (**C**), *hyfA* (**D**), and *hyfE* (**E**). Error bars represent the mean and standard deviation for three independent experiments with two technical replicates each. ** p < 0.01, and *** p < 0.001 using two-way ANOVA compared to wild-type *P. mirabilis*.

We first sought to examine the impact of co-culture on the expression of *yntA*, *hybB*, *hybC*, *hyfA*, and *hyfE* during growth in TYET (Figure 8). Expression of *yntA* was increased during co-culture with *E. faecalis* but not *P. stuartii* (Figure 8A), which could suggest increased demand for nickel import to fuel hydrogenase activity. Expression of *hybB* was also increased during co-culture with *E. faecalis* but not *P. stuartii* (Figure 8B); however, no differences were observed in *hybC* expression (Figure 8C). In contrast, both *hyfA* and *hyfE* were increased during co-culture with either *E. faecalis* or *P. stuartii* (Figure 8D,E). Thus, co-culture with either partner appears to increase expression of the Hyf system in TYET, while expression of the Hyb system is only altered during co-culture with *E. faecalis*.

**Figure 9:**
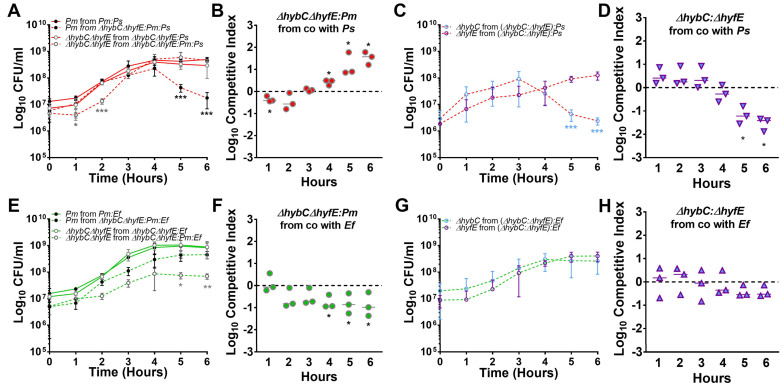
**Polymicrobial interactions alter the contribution of Hyb and Hyf to *P. mirabilis* fitness in TYET medium.** TYET medium was inoculated with a 1:1 mixture of either ∆*hybC*∆*hyfE* and wild-type *P. mirabilis* (**A**,**B**,**E**,**F**) or ∆*hybC* and ∆*hyfE* (**C**,**D**,**G**,**H**) along with either wild-type *P. stuartii* BE2467 (**A**–**D**) or *E. faecalis* 3143 (**E**–**H**), and CFUs were quantified at hourly intervals. A competitive index was again calculated using the ratio of the indicated strains at each time point compared to the inoculum (panels **B**,**D**,**F**,**H**). Data represent the mean ± SD of 3 independent experiments. Significant differences in the competitive index were determined with Wilcoxon signed rank and in growth curves using two-way ANOVA. * p < 0.05, ** *p* < 0.01, and *** *p* < 0.001.

In our TYET fitness experiments, we observed that disrupting *hybC* and the resulting increased expression of the Hyf system appeared to decrease *P. mirabilis* fitness, while disrupting *hyfE* and the resulting decreased expression of the Hyb system may increase fitness. We therefore expected *P. stuartii* coinfection to further augment the fitness advantage of the Δ*hyfE* mutant and exacerbate the defect of the Δ*hybC*, while *E. faecalis* coinfection could potentially offset the balance by increasing expression of the Hyb system. To test this hypothesis, we repeated the *P. mirabilis* mutant and wild-type co-challenge experiments in the presence of either *P. stuartii* or *E. faecalis* (Figure 9). During co-culture in TYET medium, the Δ*hybC*Δ*hyfE* double mutant exhibited an even more pronounced fitness advantage over wild-type *P. mirabilis* in stationary phase during co-culture with *P. stuartii* compared to single-species competition with wild-type *P. mirabilis* (25–40 fold increase in the ratio of mutant:wt CFUs during co-culture with *P. stuartii*, versus a 4–5 fold increase during single-species co-culture, Figure 9A,B). Importantly, the decrease in Δ*hybC*Δ*hyfE* CFUs at early time points and the decrease in wild-type *P. mirabilis* CFUs during stationary phase were both directly due to competition between *P. mirabilis* strains rather than competition with *P. stuartii*, as viability was not altered during culture of either strain alone with *P. stuartii* (Figure 9A). Similarly, no differences in *P. stuartii* viability were observed under any of the tested conditions (Supplemental Figure S2). Thus, the presence of *P. stuartii* appears to exacerbate the competition between wild-type *P. mirabilis* and the Δ*hybC*Δ*hyfE* double mutant during stationary phase in TYET medium*. P. stuartii* also appears to exacerbate the relative defect of the Δ*hybC* mutant when competed against the Δ*hyfE* mutant (Figure 9C,D), with ~15-fold decrease in fitness at 5 h and ~35-fold decrease at 6 h with *P. stuartii* compared to ~10-fold decrease at 5 h and ~14-fold decrease at 6 h during single-species competition (Figure 6G,H). Thus, *P. mirabilis* Δ*hybC* is even more strongly outcompeted by ∆*hyfE* during co-culture with *P. stuartii*, and this is likely due to the further increase in expression of the Hyf system in the Δ*hybC* mutant.

Very different fitness trends were observed with *E. faecalis.* The Δ*hybC*Δ*hyfE* double mutant was outcompeted by wild-type *P. mirabilis* when in the presence of *E. faecalis*, exhibiting a more pronounced lag phase and plateauing at a lower cell density (Figure 9E,F). The fitness defect was again directly due to altered competition between the Δ*hybC*Δ*hyfE* double mutant and wild-type *P. mirabilis* rather than competition with *E. faecalis*, as the viability of the double mutant was not altered when cultured alone with *E. faecalis* (Figure 9E). Similarly, no differences in *E. faecalis* viability were observed under any of the tested conditions (Supplemental Figure S2). Thus, flexible H_2_ metabolism provides *P. mirabilis* with an important fitness advantage during co-culture with *E. faecalis* in TYET medium. The presence of *E. faecalis* also abrogated the fitness defect of the Δ*hybC* mutant when competed with the Δ*hyfE* mutant, as both mutants grew comparably during co-culture with *E. faecalis* and no competition was observed (Figure 9G,H). Thus, the presence of *E. faecalis* not only alters expression of the Hyb and Hyf systems in *P. mirabilis* but likely shifts redox balance and H_2_ metabolism such that increased expression of the Hyf system no longer has any detrimental effect in stationary phase. 

### 3.9. Polymicrobial Interactions Alter the Contribution of [NiFe] Hydrogenases to P. mirabilis Fitness during Experimental CAUTI

**Figure 10:**
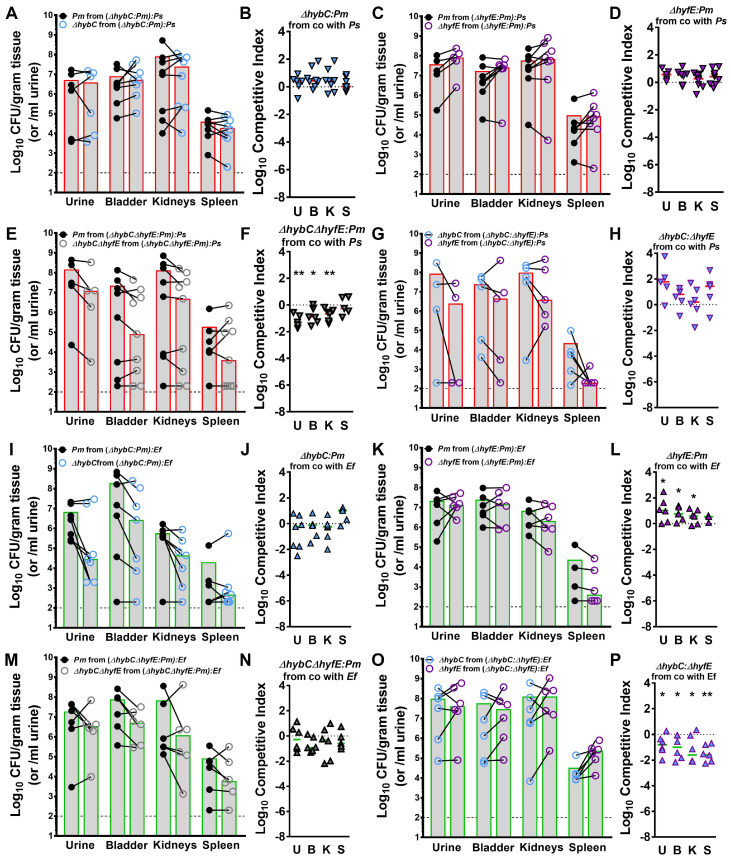
**Polymicrobial interactions alter the contribution of Hyb and Hyf to *P. mirabilis* fitness during experimental CAUTI.** Female CBA/J mice were transurethrally inoculated with 50 µL containing ∆*hybC* and wild-type *P. mirabilis* with either *P. stuartii* (**A**,**B**) or *E. faecalis* (**I**,**J**), ∆*hyfE* and wild-type *P. mirabilis* with either *P. stuartii* (**C**,**D**) or *E. faecalis* (**K**,**L**), ∆*hybC*∆*hyfE* and wild-type *P. mirabilis* with either *P. stuartii* (**E**,**F**) or *E. faecalis* (**M**,**N**), or ∆*hybC* and ∆*hyfE* with either *P. stuartii* (**G**,**H**) or *E. faecalis* (**O**,**P**). During inoculation, a 4 mm segment of sterile silicone catheter tubing was inserted into the bladder of each mouse to mimic CAUTI. (**A**,**C**,**E**,**G**,**I**,**K**,**M**,**O**) Each symbol represents the Log_10_ CFU per milliliter of urine or gram of tissue of *P. mirabilis* from an individual coinfected mouse, with the CFUs of wild-type *P. mirabilis* and hydrogenase mutants connected with a black line. Gray bars represent the mean, and the dashed line indicates the limit of detection. (**B**,**D**,**F**,**H**,**J**,**L**,**N**,**P**) A competitive index was calculated based on the ratio of each strain in each organ divided by the ratio of each strain in the input inoculum. Each symbol represents the Log_10_ CI for an individual mouse, error bars represent the median, and the dashed line indicates Log_10_ CI = 0 (the expected value if the ratio is 1:1). * *p* < 0.05, and ** *p* < 0.01 using a Wilcoxon signed-rank test.

To assess the contribution of each [NiFe] hydrogenase and overall H_2_ metabolism to *P. mirabilis* fitness during polymicrobial infection, female CBA/J mice were transurethrally inoculated as above with 10^5^ total CFUs containing a mixture of 2.5 × 10^4^ CFU of wild-type *P. mirabilis*, 2.5 × 10^4^ CFU of either ∆*hybC*, ∆*hyfE*, or the ∆*hybC*∆*hyfE* double mutant, and 5 × 10^4^ CFU of either *P. stuartii* or *E. faecalis.* The same fitness trends were observed for all three mutants during coinfection with *P. stuartii* as during single-species infection, in which H_2_ metabolism needs to be completely dysregulated with the loss of both *hybC* and *hyfE* to cause a significant fitness defect during CAUTI (Figure 10A–F). Similarly, there was no apparent competition between the ∆*hybC* and ∆*hyfE* mutants during coinfection with *P. stuartii* (Figure 10G,H). For coinfection with *E. faecalis*, none of the mutants exhibited a fitness defect (Figure 10I–N). Thus, the impact of *E. faecalis* on *P. mirabilis* H_2_ metabolism appears to alleviate the requirement for [NiFe] hydrogenases. However, disruption of *hyfE* provided a slight fitness advantage in the presence of *E. faecalis*, such that the ∆*hyfE* mutant outcompeted wild-type *P. mirabilis* (Figure 10K,L) as well as the ∆*hybC* mutant (Figure 10O,P). All fitness trends were again strictly due to competition between *P. mirabilis* and its mutants, as no differences in *P. stuartii* or *E. faecalis* CFUs were detected between infection groups (Supplemental Figure S3). Based on these data, we conclude that flexible H_2_ metabolism contributes to *P. mirabilis* fitness during single-species CAUTI, but the presence of different coinfection partners alters the redox landscape and the subsequent contribution of the HyB and Hyf systems.

## 4. Discussion

The majority of studies pertaining to *P. mirabilis* pathogenesis in the urinary tract have focused on defining fitness and virulence factors for single-species infection [5]. However, prospective longitudinal studies have demonstrated that *P. mirabilis* often persistently colonizes the catheterized urinary tract as part of a polymicrobial community [4,16]. Using genome-wide screens such as transposon insertion-site sequencing, we have found that microbe–microbe interactions dramatically alter the genes and pathways required by each constituent to establish infection, including factors that were previously thought to be strong candidates for vaccine development and therapeutics [15,41]. Thus, examining the impact of common coinfection partners on fitness requirements is an important triage step for identifying the ideal targets for therapeutics and vaccine development against bacterial species that are commonly part of a polymicrobial infection. 

In this study, we determined that disruption of either *hybC* or *hyfE* alone had no impact on *P. mirabilis* fitness during experimental CAUTI. However, the combined disruption of both genes and resulting loss of flexible H_2_ metabolism substantially decreased *P. mirabilis* fitness within the urinary tract during single-species infection and during coinfection with *P. stuartii*, but not during coinfection with *E. faecalis.* Considering that *E. faecalis* is the most common and persistent co-colonization and coinfection partner of *P. mirabilis* in the catheterized urinary tract [4,42], our data suggest that neither [NiFe] hydrogenase system would be a strong therapeutic target against *P. mirabilis*.

The lack of a fitness defect for the *hyfE* mutant during CAUTI was surprising considering that disruption of *hyfG* in a different strain of *P. mirabilis* resulted in decreased colonization in a mouse model of uncomplicated UTI [21]. This discrepancy could be due to differences in the strain used, the gene that was disrupted, the use of a co-challenge model of assessing fitness compared to an independent challenge model, or the specific infection model. The presence of a urinary catheter induces a potent inflammatory response even in the absence of colonizing microbes [43,44], and this substantially alters urine composition and likely also influences the redox landscape compared to an uncomplicated UTI. 

Considering that *hyfE* was the only gene pertaining to the Hyf-type Group 4a [NiFe] hydrogenase that was identified as a candidate fitness factor for CAUTI via TnSeq and that it was only a 3.6-fold defect [15], our experimental data indicate that this was a false-positive and that the Hyf-type hydrogenase does not contribute to *P. mirabilis* fitness during CAUTI. The lack of fitness defect during polymicrobial infection with *P. stuartii* was even more surprising, considering that six genes in the *hyf* operon were identified as candidate fitness factors under this condition [15]. However, all six genes exhibited less than a 4-fold defect via TnSeq, suggesting that the criteria for estimation of conditionally essential genes may not have been stringent enough. This would also explain the lack of fitness defect for the *hybC* mutant during coinfection with *P. stuartii*, as the largest defect detected via TnSeq was 6.4-fold (*hybE*) and the rest of the genes exhibited less than a 4-fold defect. 

One of the most striking observations was the shift in in vitro fitness of the ∆*hybC*∆*hyfE* double mutant and the lack of an in vivo fitness defect during coinfection with *E. faecalis.* This is particularly notable since disruption of both *hybC* and *hyfE* altered transcriptional regulation of both [NiFe] hydrogenases in vitro and resulted in a pronounced fitness defect during single-species infection as well as coinfection with *P. stuartii*. Taken together, our data strongly suggest that the presence of *E. faecalis* alters the redox landscape both in vitro and within the catheterized urinary tract. 

Finally, the observation that both *hybC* and *hyfE* contributed to benzyl viologen reduction in TYET and that disruption of either gene altered expression of the other system was unexpected and hints at complexity in the TYET assay. Further study is needed to fully characterize H_2_ oxidation and H_2_ production in *P. mirabilis*. However, our data clearly indicate that polymicrobial interactions alter H_2_ metabolism. Considering that CAUTI is typically polymicrobial and can involve concurrent colonization with 2–5 species, our findings highlight the importance of defining a core set of fitness factors required by each species regardless of their coinfection partners.

## Data Availability

All data that support the findings of this study are available in the main text, Supplementary Materials, or deposited in appropriate repositories. All strains will be freely shared by the corresponding author upon request.

## Supplementary Materials

The following supporting information can be downloaded at https://www.mdpi.com/article/10.3390/pathogens12121377/s1, Figure S1. HybC and HyfE do not contribute to fitness during aerobic growth in human urine in vitro. Pooled human urine was inoculated with a 1:1 mixture of ∆*hybC* and wild-type *P. mirabilis* (A,B), ∆*hyfE* and wild-type *P. mirabilis* (C,D), ∆*hybC*∆*hyfE* and wild-type *P. mirabilis* (E,F), or ∆*hybC* and ∆*hyfE* (G,H) and CFUs were quantified at hourly intervals. A competitive index was calculated using the ratio of mutant/wt at each time point compared to the ratio at time = 0 (panels B,D,F,H). The data represent the mean ± SD of three independent experiments. No significant differences in the competitive index were determined with Wilcoxon signed rank. * *p* < 0.05 using two-way ANOVA. Figure S2. Co-culture with *P. mirabilis* hydrogenase mutants does not alter viability of *P. stuartii* or *E. faecalis*. The TYET medium was inoculated with *P. stuartii* (A) or *E. faecalis* (B) along with wild-type *P. mirabilis*, the ∆*hybC*∆*hyfE* double mutant, or a 1:1 mixture of ∆*hybC*∆*hyfE* and wild-type *P. mirabilis* and CFUs were quantified at hourly intervals. Data represent mean ± SD of three independent experiments. No significant differences were detected using two-way ANOVA. Figure S3. Comparison of *P. mirabilis*, *P. stuartii*, and *E. faecalis* CFUs across coinfections. Each symbol represents the Log_10_ CFU per milliliter of urine or gram of tissue for *P. mirabilis* (A), *P. stuartii* (B), *E. faecalis* (C), ∆*hybC* (D), ∆*hyfE* (E), and ∆*hybC*∆*hyfE* (F) across each coinfection. Gray bars represent the median, and dashed lines indicate the limit of detection. ** p* < 0.05, and *** p* < 0.01 with a non-parametric Kruskal–Wallis test.
