# Supplementary figures and images for: Differential Contribution of Hydrogen Metabolism to Proteus mirabilis Fitness during Single-Species and Polymicrobial Catheterized Urinary Tract Infection
Source: Pathogens. 2023 Nov 22;12(12):1377. doi: 10.3390/pathogens12121377 (PMC10745698; doi:10.3390/pathogens12121377)

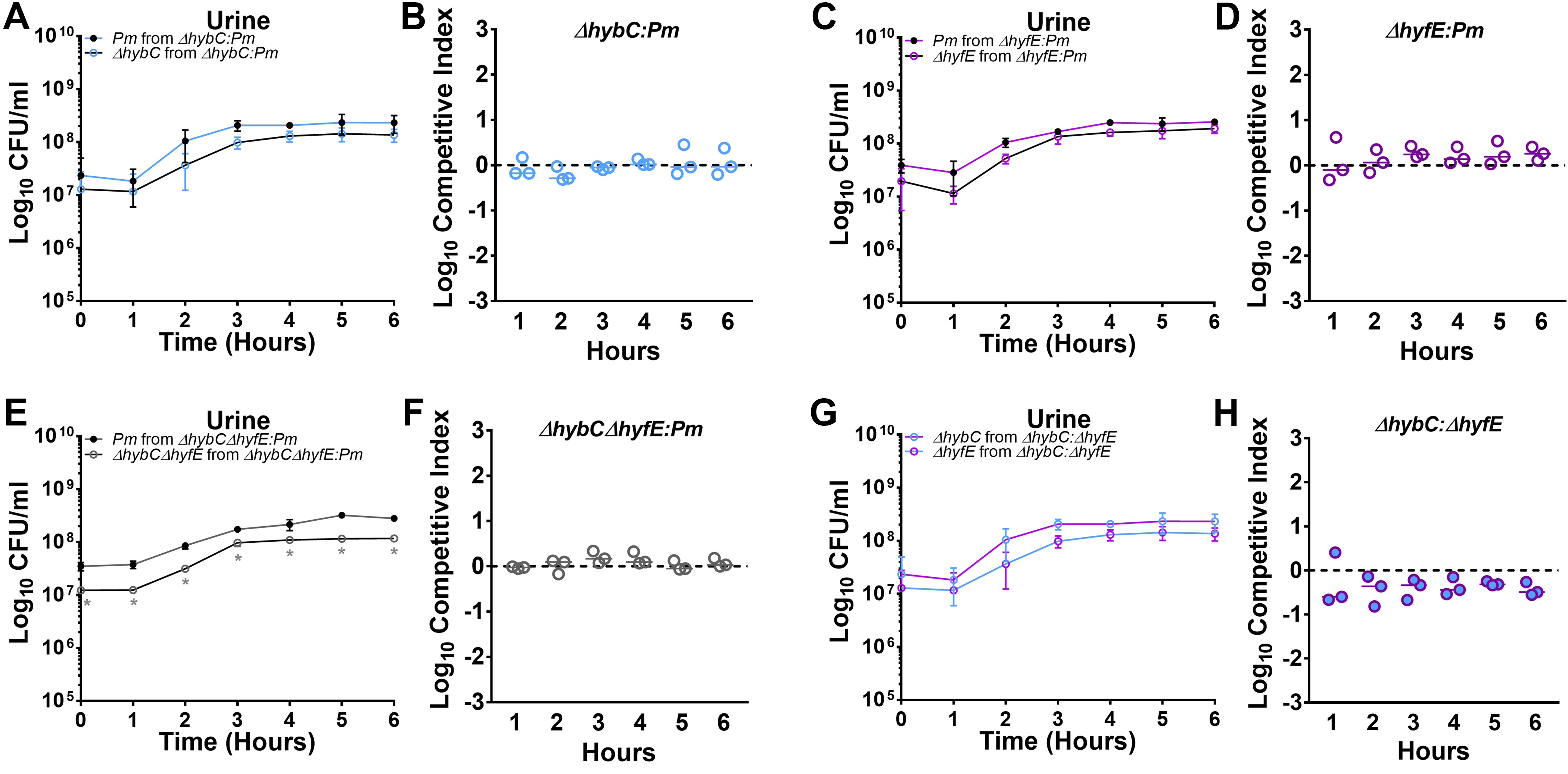

Supplement: Supplementary file 1 [file pathogens-12-01377-s001.zip › Supplemental Figure S1.tif]

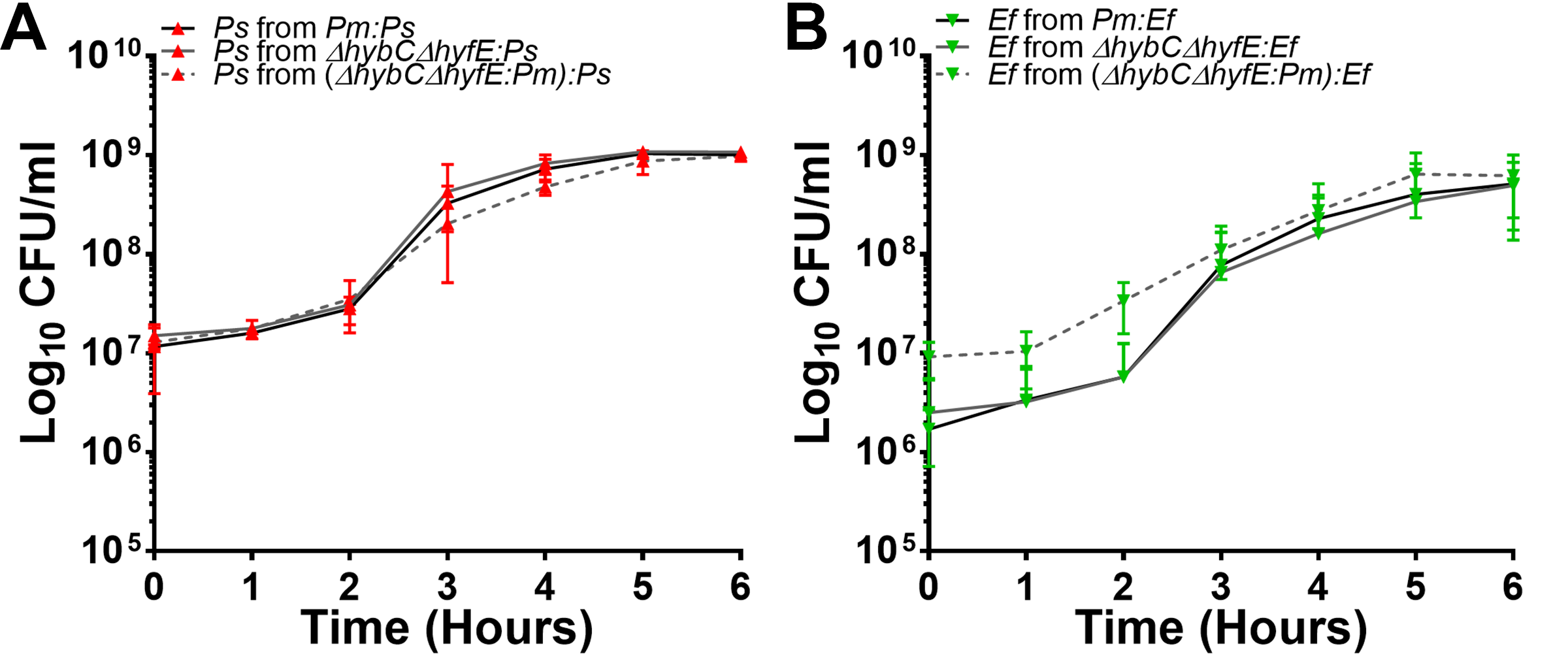

Supplement: Supplementary file 1 [file pathogens-12-01377-s001.zip › Supplemental Figure S2.tif]

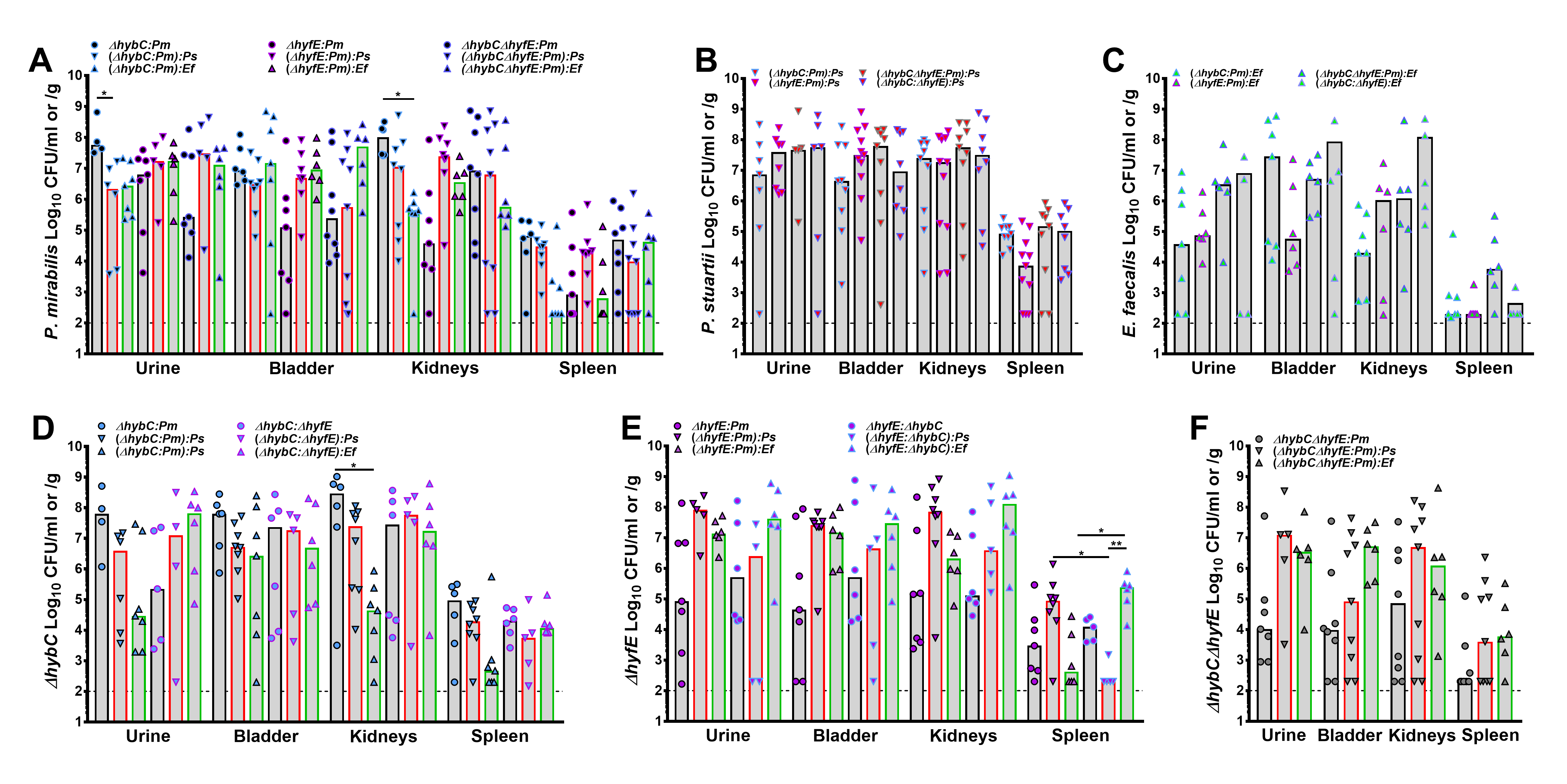

Supplement: Supplementary file 1 [file pathogens-12-01377-s001.zip › Supplemental Figure S3 revised.tif]
